# Role of the PD-1 and PD-L1 axis in COVID-19

**DOI:** 10.2217/fmb-2022-0103

**Published:** 2022-07-27

**Authors:** Srinivasa R Bonam, Haitao Hu, Jagadeesh Bayry

**Affiliations:** ^1^Department of Microbiology & Immunology, University of Texas Medical Branch, Galveston, TX 77555, USA; ^2^Institute for Human Infections & Immunity, Sealy Institute for Vaccine Sciences, University of Texas Medical Branch, Galveston, TX 77555, USA; ^3^Department of Biological Sciences & Engineering, Indian Institute of Technology Palakkad, Palakkad, 678623, India

**Keywords:** adaptive immune cells, COVID-19, immune checkpoint molecules, Innate immune cells, PD-1, PD-L1, SARS-CoV-2

## Abstract

Severe COVID-19 patients display dysregulated expression of checkpoint molecules PD-1 and its ligand PD-L1, suggesting that these checkpoint molecules could be considered as prognostic markers and therapeutic targets in severe cases of COVID-19.

PD-L1 is a transmembrane protein with a molecular weight of 40 kDa and it signals through a short cytoplasmic tail. Synonymously, it is also termed as B7-H1 (a member of B7 family) and CD274. Numerous cell types express the PD-L1, including leukocytes (granulocytes, monocytes, macrophages, dendritic cells, mast cells, basophils, platelets, B cells, T cells and others), nonhematopoietic cells (cancer cells, microglia, astrocytes, neurons and epithelial cells) and nonlymphoid cells (muscle, heart, placenta and renal tubular cells) [1]. PD-L1 and PD-L2 were discovered in 2000 and 2001, respectively, as negative regulators of T-cell activation [2]. Compared with PD-L1, its putative receptor, PD-1 (CD279; 55 kDa) expressed on the surface of antigen-stimulated T cells, was discovered earlier in 1992. Since its discovery, several studies have confirmed the role of PD-1 in the maintenance of immune tolerance. Later findings identified the role of PD-L1 in protecting the host from autoimmunity by maintaining self-tolerance with the help of regulatory T cells (Treg cells). On the other hand, PD-1/PD-L1 axis limits anti-tumor immunity and therapeutic response. Blocking the abovementioned signaling pathway has shown beneficial clinical outcomes in several cancer types. Therefore, PD-L1 expression has been considered as a predictive biomarker for cancer immunotherapy, although dynamic and heterogeneous expression of PD-L1 is a limiting factor.

Several recent reports also highlight that PD-1/PD-L1 axis plays a role in the pathogenesis of various infectious diseases, including AIDS and hepatitis B. Though in the acute condition, the interaction between the checkpoint molecules PD-1 and its ligand PD-L1 help in reducing the infection-associated inflammation and inflammation-mediated tissue damage, chronic stimulation leads to immune exhaustion, reduced effector functions of immune cells and progression of the disease as reported in case of the recent COVID-19 caused by SARS-CoV-2. Therefore, therapeutically blocking this pathway ‘transiently’ would activate the immunity against pathogens.

In general, innate immunity lowers the viral load and adaptive immunity, particularly T cells, clear the virus-infected cells. For various reasons, SARS-CoV-2 hijacks the innate immune defense system and induces immune checkpoint molecules. When SARS-CoV-2 infects lung epithelial cells, they produce inflammatory cytokines, like IL-6 and TNF-α. In addition, initial interaction with the virus leads to PD-L1 expression on the epithelial cells. The reason for the expression of this molecule, either ‘eat me signal’ or ‘save me signal’ is not known yet. The secreted cytokines recruit the innate immune cells (first neutrophils and then monocytes) to the location, followed by the activation of innate immune cells. More than epithelial cells, innate immune cells secrete copious amounts of proinflammatory cytokines (IL-6, IL-8, TNF-α and others; denoted as cytokine storm) followed by the recruitment of adaptive immune cells.

Studies on the gene expression of *PDL1* from *in vitro* and *ex vivo* experiments have revealed more interesting facts. Lung epithelial cells (Calu3 and A549 [transduced with ACE2]) following incubation with SARS-CoV-2 have shown elevated levels of PD-L1 [3]. Supporting the above data, lung biopsies of COVID-19 patients were tested for *PDL1* gene expression, revealing upregulation of PD-L1 expression compared with healthy donors [3].

## Dysregulated surface expression of PD-1/PD-L1 in innate immune cells & T cells of COVID-19 patients

Several reports have now demonstrated that severe and critical COVID-19 patients display dysregulated PD-1/PD-L1 axis. The expression of PD-L1 is enhanced on both dendritic cells (myeloid and plasmacytoid dendritic cell subsets) and monocytes in severe COVID-19 patients [4,5] and was associated with their inability to respond to stimuli [5] and reduced T cell stimulatory capacity [6]. Though the functional consequences are not yet clear, disease severity also appears to be associated with the emergence of PD-L1 expression in eosinophils and basophils [7]. In addition, platelets from COVID-19 patients show an increased expression of PD-L1 that negatively affects the CD4^+^ T cell IFN-γ production when cocultured with the platelets [8].

On the other hand, T cells from the severe COVID-19 patients show an exhausted phenotype, functional paralysis and an enhanced expression of PD-1 and other checkpoint molecules like TIGIT (T cell immunoreceptor with immunoglobulin and ITIM domain) and TIM-3 (T cell immunoglobulin and mucin-domain containing-3) that indicates the progression toward immunosuppressive milieu [9–11]. Furthermore, extended epigenetic studies on the T cells have revealed altered degree of *PD1* promoter methylation in CD8^+^ T cells [12]. Nonetheless, the precise role of PD-1/PD-L1 axis in the pathogenesis of COVID-19 is far from clear.

Enhanced expression of PD-L1 on the innate cells of COVID-19 patients could be due to both SARS-CoV-2 and/or inflammatory milieu induced by the virus infection. Under *in vitro* conditions, stimulation of plasmacytoid dendritic cells with SARS-CoV-2 has been reported to induce surface expression of PD-L1 [13]. Cytokines like IFN-γ that are induced due to host response to SARS-CoV-2 are also capable of inducing PD-L1. We found that SARS-CoV-2 is incapable of inducing PD-L1 on human basophils [14]; however, under IL-3 priming conditions, IFN-γ could induce it [15]. Hypoxia-induced multiple organ injury could also enhance the expression of PD-L1 [16].

## Elevated levels of soluble forms of PD-L1 in COVID-19 patients

Interestingly, recent studies have highlighted the existence of different forms of PD-L1, such as a surface of plasma membrane, the surface of exosomes, cell nuclei and circulating soluble PD-L1. However, the underlying mechanisms on their generation are not fully characterized [17]. One of the recent studies has characterized both soluble and genomic expression of PD-L1 in COVID-19 patients. It was found that soluble PD-L1 levels were significantly elevated in the blood of COVID-19 patients compared with healthy donors [3]. The reason for the shedding and enhanced soluble levels of PD-L1 in COVID-19 patients is not known. SARS-CoV-2 could act as one of the triggering factors as a direct correlation between viral RNA load in the plasma of the patients and the soluble PD-L1 has been reported [18].

## PD-L1 as a potential biomarker of COVID-19

Circulating levels of soluble PD-L1 has been proposed as a promising biomarker to predict the severity of the disease and to identify the COVID-19 patients that need invasive mechanical ventilation [19]. Further, soluble PD-L1 in COVID-19 patients is positively correlated with increased C-reactive protein and negatively correlated with decreased lymphocytes, PaO_2_/FIO_2_ (arterial oxygen partial pressure/fractional inspired oxygen). Similarly, increased plasma levels of soluble PD-1 and PD-L1 in COVID-19 patients is associated with decreased immune response of peripheral blood mononuclear cells to nonspecific antigens [12].

## PD-1/PD-L1 axis as a therapeutic target in COVID-19: rationale & evidence

As discussed above, targeting ‘transiently’ (to avoid the adverse effects of long-term inhibition) the PD-1/PD-L1 axis or its pathways of induction in the early stages of infection might have a beneficial outcome. Furthermore, under the therapeutic setting, kinase inhibitors could suppress the PD-L1 expression. Ruxolitinib, a reversible and selective JAK1 and JAK2 inhibitor, has been shown to reduce the PD-L1 expression in lung epithelial cells that have been exposed to SARS-CoV-2 [3].

It has been documented that during the viral replication process, cells secrete anti-inflammatory cytokine, IL-10, that regulates the expression of PD-L1 and PD-1 in a STAT-3-dependent pathway on dendritic cells/monocytes and T cells, respectively [1,20]. In addition, several studies have reported elevated levels of IL-10 in the COVID-19 patients. Therefore, either blocking IL-10 or blocking both IL-10 and PD-L1, serves as viable targets for therapeutic purposes in chronic infections. Of note, blockade of PD-L1 could restore the *ex vivo* IFN-γ-stimulatory capacity of platelets in COVID-19 patients [8]. Also, *ex vivo* blockade of PD-1 has re-established T cell function of COVID-19 patients [12]. That being said, none of the interesting clinical studies has been registered in these directions.

There are several outstanding questions that need to be answered in the future, including detailed investigation of checkpoint molecules and kinetics of expression in various immune cells of COVID-19 patients, the mechanisms of their induction and shedding, proof of concept on their use as therapeutic targets and the genetic basis like variants in the genes and epigenetic factors that lead to dysregulated expression of checkpoint molecules in the COVID-19 patients.
